# iPS Cells—The Triumphs and Tribulations

**DOI:** 10.3390/dj4020019

**Published:** 2016-06-06

**Authors:** Riddhi Sharma

**Affiliations:** Department of Craniofacial Development and Stem Cell Biology, Dental Institute, King’s College London, London SE1 9RT, UK; riddhi.sharma@kcl.ac.uk or dr.riddhi.sharma@gmail.com; Tel.: +91-702-220-6333

**Keywords:** human induced pluripotent stem cells, iPS cells, pluripotency, stem cells, regeneration, therapeutic potential, regenerative dentistry, clinical trials, dental stem cells, regenerative medicine

## Abstract

The year 2006 will be remembered monumentally in science, particularly in the stem cell biology field, for the first instance of generation of induced pluripotent stem cells (iPSCs) from mouse embryonic/adult fibroblasts being reported by Takahashi and Yamanaka. A year later, human iPSCs (hiPSCs) were generated from adult human skin fibroblasts by using quartet of genes, Oct4, Sox2, Klf4, and c-Myc. This revolutionary technology won Yamanaka Nobel Prize in Physiology and Medicine in 2012. Like human embryonic stem cells (hESCs), iPSCs are pluripotent and have the capability for self-renewal. Moreover, complications of immune rejection for therapeutic applications would be greatly eliminated by generating iPSCs from individual patients. This has enabled their use for drug screening/discovery and disease modelling *in vitro*; and for immunotherapy and regenerative cellular therapies *in vivo*, paving paths for new therapeutics. Although this breakthrough technology has a huge potential, generation of these unusual cells is still slow, ineffectual, fraught with pitfalls, and unsafe for human use. In this review, I describe how iPSCs are being triumphantly used to lay foundation for a fully functional discipline of regenerative dentistry and medicine, alongside discussing the challenges of translating therapies into clinics. I also discuss their future implications in regenerative dentistry field.

## 1. Introduction

The discovery of Embryonic Stem Cells (ESCs) [1,2,3] incited the search for discovering artificial differentiation techniques to confer the properties of ESCs onto somatic cells by altering epigenomic activity, such that the derived cells are pluripotent and capable of giving rise to embryonic-like stem cells. These techniques are collectively referred to as cellular reprogramming. Yamanaka’s pioneering experiments made it possible in 2006 to create embryonic-like stem cells from mouse fibroblast cultures, without using embryos by engineering his creation *in vitro* [4].

**Figure 1:**
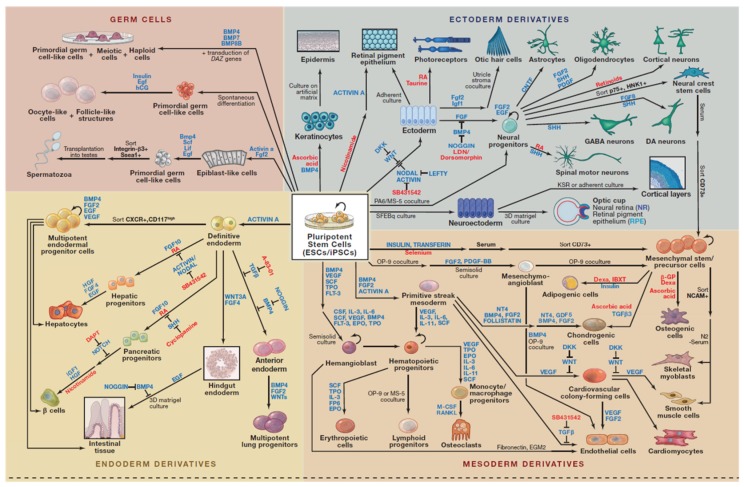
Directed Differentiation of Pluripotent Stem Cells [8]. Highlighted here are some of strategies for directing the differentiation of Embryonic Stem Cells (ESCs) and induced pluripotent stem cells (iPSCs) into defined cell types. Most cell types and pathways depicted correspond to published work on human cells, expect for the production of spermatozoa, oocyte-like cells, otic hair cells, cortical layers, and optic cup, which were generated with mouse ESCs or iPSCs. This figure is reproduced from Williams, Davis-Dusenbery and Eggan [8]; published by Elsevier under open-access license policies.

Human induced pluripotent stem cells (hiPSCs) were generated by using either by retroviral transduction of fibroblasts encoding the original four transcription factors, constitutively expressed the POU domain class 5 transcription factor 1 (Oct3/4), the sex determining region Y-box2 (Sox2), Kruppel-like factor 4 (Klf4), and myelocytomatosis oncogene (c-Myc) also referred as OSKM—“Yamanaka’s cocktail” [5], or by independently determined combination of lentivirally transduced genes Oct3/4, Sox2, NANOG (Nanoghomeobox), and Lin28 [4,6,7]. While these reprogrammed cells have similar developmental potential as authentic hESCs, they come without the baggage of morality and ethics, as they are not derived from human embryos and the possibility of immune rejection from allogeneic transplantation. In addition, these hiPSCs resemble hESCs in their morphology and gene expression and can differentiate into cell types of all the three primary germ layers (ectoderm, endoderm and mesoderm) *in vitro* and *in vivo* (Figure 1).

In this review, I present a comprehensive overview of factors playing role in generation of iPSCs and the present day cellular reprogramming alternatives. I will discuss applications and advantages of iPSCs followed by challenges associated with their clinical applications. In the end, I will briefly discuss the future prospects of iPSCs in the field of regenerative dentistry.

## 2. Factors of Importance in the Generation of iPSCs

**Figure 2:**
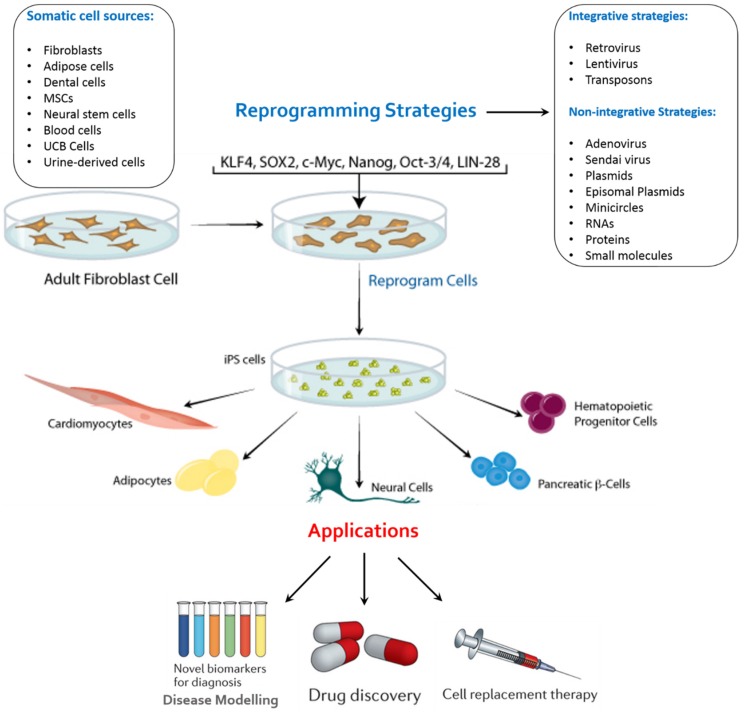
An overview of key reprogramming methods available for the generation of iPSCs from various somatic cell sources and their possible applications. Adult stem cells or iPSCs can be expanded in culture and differentiated into the disease-affected cells that can be used to recapitulated disease pathogenesis *in vitro.* Patient-specific disease models can be used to identify new biomarkers for improved diagnostic procedures, such as earlier detection of disease onset. These disease models can also be used to identify compounds that alleviate disease pathology *in vitro*, which can be further developed into novel drugs. Such compounds can be identified by carrying out a phenotypic screen using the disease model to identify compounds that may act through novel mechanisms. Stem-cell-derived cells can also form the basis for cell replacement therapies.

The reprogramming factors have their individual role and at the same time, they interact with each other complimentarily. Two methods for delivering the reprogramming transcription factors into the somatic cells are, Integrating Viral Vector Systems and Non-integrating Systems (Figure 2). The viral vector gets integrated into host genome in case of integrating methods. The use of retrovirus and lentivirus falls into this category. However, long-term safety of hiPSCs cannot be assured through mouse studies alone. In addition, even though this method is highly efficient, there is a risk of multiple chromosomal disruptions, any of which may cause genetic dysfunction and/or tumorigenesis. In addition, retroviruses may make iPSCs immunogenic [9]. Thus, we will need to avoid induction methods that involve vector integration into the host genome for the purpose of cell transplantation therapy and hence, altered methodologies have been toiled upon. In non-integrating methods, there is no integration in the host cell genome. The use of Viral vectors like the Adeno virus [10] and Sendai virus [11], plasmid DNA [12,13], synthesized mRNAs [14] and proteins [15] fall under this category. Plasmids such as oriP/EBNA1 (derived from Epstein-bar virus) have been used for reprogramming but they have demonstrated to be of low efficacy [16]. Direct delivery of reprogramming proteins has also been carried out by fusing them with a cell penetrating peptide [15]. A different approach using a single self-replicating RNA replicon, which expressed high levels of Yamanaka factors for transfection into fibroblasts to be reprogrammed into iPSCs, was used and iPSCs displayed all properties of pluripotent stem cells [17]. Finally, small-molecule drugs have been investigated for establishing safe methods of iPSC generation for clinical application because they are non-immunogenic, cost-effective, and easy to handle [18]. Recently, successful reprogramming of mouse somatic cells without transgene introduction was achieved with small-molecule drug combinations [19].

Human skin fibroblasts were used as a starting material for generation of iPSCs and are the major source due to their commercial availability and ease of gene-delivery. However, the process is lowly efficient as typically less than 1% of transfected fibroblasts become iPSCs. In addition, fibroblasts are not amenable to large-scale production due to the technical challenges of establishing stable fibroblast cell lines and the need for invasive skin biopsies to obtain human tissue. iPSCs can also be derived from keratinocytes, mesenchymal cells, adipose stem cells, melanocytes [20] and postmitotic neurons [21].

A more desirable source is human peripheral blood as is easily obtainable through routine, non-invasive clinical procedures. Their use also enables the creation of iPSCs from large number of donor samples stored in biorepositories worldwide. Efforts to develop such methods have yielded iPSCs from human CD34+ blood cells and T-lymphocytes [22,23,24,25]. It was revealed in a particularly key study that hiPSCs can be efficiently derived from T lymphocytes from as little as 1 mL of whole blood [26]. Thus, most, if not all, somatic cells have a potential to become iPSCs, albeit with different efficiencies [27].

## 3. A Novel Culture System for iPSCs Derivation

In order to use hiPSCs in regenerative medicine, the cells must be prepared using methods compliant with GMP (Good Manufacturing Practice). However, the methods in use up till now use feeder cells to culture iPSCs, which involve complex procedures, and employ culture medium containing serum and numerous other animal-derived constituents, which make it difficult for them to comply with GMP and hence unsuitable for regenerative therapies in humans. Chen *et al.* [28] recently reported the development of a significantly improved hiPSC culture medium, TeSR^™^-E8^™^, which contains only eight completely defined and xeno-free (free of animal-derived constituents) components. TeSR^™^-E8^™^ is based on the E8 formulation published by Dr. James Thomson, the lead researcher behind the mTeSR^™^1 formula [29,30] and contains a minimized set of the components required for maintenance of hiPSCs, providing a simpler medium. This medium is low in protein compared to other conventional feeder-free culture medium such as mTeSR^™^1 and TeSR^™^2. Beers *et al.* [31] described an EDTA-based, enzyme-free passaging protocol for routine maintenance and reprogramming of iPSCs, which achieves maximum cell survival without enzyme neutralization, centrifugation or drug treatment. Wang *et al.* [32] demonstrated an efficiently scalable culture system using the E8 for the expansion and cryopreservation of hiPSCs under adherent and suspension culture conditions. Another method for generation and maintenance culture of iPSCs that are suitable for use in cell transplantation therapy has been recently developed [33]. They established that recombinant laminin-511 E8 fragments are useful matrices for maintaining hiPSCs when used in combination with a completely xeno-free medium, StemFit^™^. Using this system, hiPSCs can be easily and stably passaged by dissociating the cells into single cells for long periods, without any chromosomal/karyotype abnormalities. hiPSCs could be generated under feeder-free and xeno-free culture systems from human skin fibroblasts and blood cells, and they possessed differentiation abilities. These results indicate that hiPSCs can be generated and maintained under this novel culture system making them ideal for cell transplantation in humans. In fact, the cell production company Lonza has generated current GMP-qualified xeno-free hiPSC lines from peripheral blood mononuclear cells, which are now available for clinical applications [34].

The microenvironment is also an important factor for consideration when developing a reliable and GMP-compliant cell culture system for regenerative therapies. Matthias Lutolf and colleagues have recently described a 3D culture system that promotes iPSC generation by modulating microenvironmental stiffness, degradability and biochemical composition [35]. They show that exposure to a 3D microenvironment enhances cell reprogramming compared to the traditional 2D environment suggesting that the 3D microenvironment keeps cells in an active proliferation state throughout the reprogramming process and that induction of iPSCs might prove to be faster in 3D conditions. They also show that with respect to a 2D system, the physical cell confinement imposed by the 3D microenvironment can increase the reprogramming efficiency of both mouse and human iPSCs by more than two-fold and, as with a recent 2D culture system, this occurs via an accelerated mesenchymal-to-epithelial transition and increased epigenetic remodelling. 3D models are also able to incorporate the spatial and structural features of tissues and/or organs affected by diseases and thereby could reveal more relevant information. For example, one study recapitulated the pathological phenotypes of Alzheimer’s disease with a 3D model [36]. Researchers have already generated 3D cardiac tissues from hiPSCs that successfully model the human heart [37]. Recently, they also developed organoids with iPSCs that mimic the cerebrum [38].

## 4. Applications of iPSCs

The treatment of multiple diseases is challenging/impossible because of inadequate information is available on the mechanisms involved in the disease progression. To be able to develop the treatment aiming at the root of the disease, diseases need to be modelled. There are already large numbers of disease testing models available but only few of them are adept at simulating human cellular microenvironment and metabolism to some extent. Animal models of rat, mice, monkey, dog, and primates have been used for disease modelling until now. However, their use is limited due to existing variability in the genetic make-up of them, which is mainly accountable for the biological functions, and hence metamorphoses are exhibited compared to humans. Thus, an altered approach is desired which can offer identical environment as human cells and iPSCs seem like an amenable substitute with plethora of advantages. iPSCs do not require multiple proliferation and their derivatives are functional after transplantation *in vitro* as well as *in vivo* [39,40,41,42,43]. Hence iPSCs are used in therapeutics for disease modelling, drug screening and regenerative medicine. Another significant use of iPSCs is to generate differentiated cells that are unavailable from adult sources that can integrate into the recipient and replace the damaged or missing cells. Examples of such therapies include retinal pigment epithelial (RPE) cell replacement in macular degeneration, making liver cells to treat liver cirrhosis, the generation of dopaminergic neurons to treat Parkinson disease, or deriving cardiac myocytes or pancreatic islets to treat cardiac disease or diabetes. In regenerative dentistry, iPSCs can be utilised for oral tissue regeneration and development of clinical treatments for the congenital diseases.

### 4.1. Disease Remodelling

We can group human diseases into three broad categories: genetic, epigenetic and acute environmental [44]. Modelling of all three types is possible *in vitro* using stem cells, and an excellent way to study the intricate mechanisms and pathways underlying the aetiology and pathophysiology of disease. Stem cells in general are ideal for creating “disease-in-a-dish” models because of their capacity for self-renewal and differentiation, their potential for recapitulating disease pathogenesis, and also their amenability for developing and testing therapeutics [45].

Using patient-specific iPSCs we can recapitulate the suspected effects of the environmental or epigenetic component known to have contributed to the patient’s disease in order to understand its severity or mechanism of action before, during, or after reprogramming. Such models can help us gain better insight into the environmental or epigenetic factors affecting a complex disease in terms of susceptibility, prognosis as well as outcomes. Patient-specific models can also be used as special models, as they can involve known epigenetic changes contributing to the disease. iPSCs can also be used for modelling disease at the organ level as well as understanding systemic diseases.

Multiple diseases have been successfully modelled using iPSCs, for example, Alzheimer’s disease [46,47], Parkinson’s disease [48,49], amyotrophic lateral sclerosis (ALS) or Lou Gehrig’s disease [50,51,52], Huntington disease [53,54], Downs syndrome/trisomy 21 [55], Type 1 diabetes mellitus [56], *etc.* Blood disorder disease modeling has been focused on sickle cell anaemia and leukemia [57,58,59,60,61]. The cell lines developed for disease modelling were also found to be consistent phenotypically, which is necessary for disease modelling. Fong *et al*. developed Tauopathy derived iPSCs carrying a TAU-A152T mutation and the phenotypes they observed in the cells from iPSCs were consistent with those in patients with the mutation [62]. There are many human diseases, which have not been recapitulated in small animal models, especially in adult onset diseases [63].

### 4.2. Drug Screening

iPSCs can be beneficially used for drug discovery and cytotoxic studies in humans. *In vitro* animal derived cells have been used as testing systems up until now but their incompetence in replicating the “exact” human physiological environment and related phenotypic attributions renders their usage. Sometimes, the benefits proven in the animal models do not turn out to be of value in humans at all. In addition, animal models are inappropriate for testing the drug toxicity as the chemical toxicity differs from animal to animal and the same applies to carcinogenic agents. Finally, a newly discovered drug or therapy must be tested on human cells or test models before being introduced in market so that the results can be concluded for safe administration in humans. These studies include steps such as prediction/identification of a potential drug molecule followed by its synthesis, generation of iPSCs, their differentiation to specific somatic cells, and testing for toxic or non-toxic effects of the synthesized drug on the somatic cells. For toxicity studies, iPSCs from normal and diseased cells are differentiated to specialized cell types.

Only 10% of the drugs that enter clinical trials are able to reach the market approval stage. The cost of developing a drug is increasing with the estimated cost of whole process being US $1.2–1.7 billion per drug compound [64,65,66]. The development of 30% of the medicines was abandoned because of lack of efficacy and 30% due to safety concerns (cardiotoxicity, hepatotoxicity) [67]. The process becomes costlier and extensively time consuming due to early detection failure of drug toxicity in human tissues. Development of toxicity models that predict more precisely before starting the clinical trials may help cut costs and time by demonstrating cardiotoxicity, hepatotoxicity or cytotoxicity caused by the drugs.

iPSCs have been explored by many research groups as their use offer better substitute to conventional tests and better chemical safety assessment as they provide similar environment to human physiological conditions than conventional animal testing models. For example, pluripotent stem cells have been used previously to establish test systems for cytotoxicity [68].

The variability and possibly incomplete reprogramming of iPSCs from somatic cells remain a considerable challenge in the integration of iPSCs into drug discovery. The process of reprogramming involves TET enzymes that mediate the generation of 5-hydroxymethylcytosine (5hmC). A study comparing the activities of TET enzymes in hESCs and hiPSCs suggests that hiPSCs represent an incompletely reprogrammed pluripotent state. This difference in 5hmC detected between hESCs and hiPSCs is also present between clones of hiPSCs, which may account for the variation between hiPSC lines [69]. This variability will likely affect the reproducibility of the results from these studies making it important to develop robust differentiation protocols and reliable assessments of the functionality of a hiPSC-derived disease model before fully integrating these cells into the process of drug development.

As iPSCs can be derived from individual patients, these offer scientists an opportunity for modelling diseases on a patient-by-patient basis. This enables screening the genomic differences between individuals that may help in the progression of disease, and the screening of pharmacological agents to find the ideal one for each individual [70]. During the drug screening procedure, hepatocytes play a central role due to their detoxification capacity. The generation of foetal hepatocyte-like cells from iPSCs resulted in differentiated cells that did not display the functions of fully mature hepatocytes and their viability after cryopreservation is extremely variable [71] which is a major drawback for iPSC-derived drug screening platforms. However, Zhang *et al*. [72] showed that hiPSC-derived mature hepatocyte-like cells hardly ever proliferated *in vitro*, and in contrast, hiPSC-derived hepatic endoderm cells exhibited a marked proliferative capability. Due to the lack of standardization of protocols, the amount of available mature hepatocytes have been insufficient for clinical therapy and drug development in the past several decades [73].

### 4.3. Regenerative Medicine

In regenerative medicine, the injured/degenerated tissues are repaired by generating those tissues with the help of iPSCs in labs and then transplanting them to the site of injury/degeneration. The core concerns with the current transplantation therapy are availability of organ or tissue and immunorejection. There is ever increasing need for organs but quite a shortage of donors, which leads to death of the patients suffering from degenerative disease or accident stricken. In addition, the cell, tissue or organ transplant is only possible from disease-free and physiologically matching donor profile with the patient, hence multiple tests are necessary before accepting/transplanting the donor tissue or organ. The iPSCs are our best bet for the reason being transplanted cells will be differentiated from the repaired iPSCs generated from patient’s own somatic cells. Once the specific cells are formed, they can be directly transplanted to the specific site to cure the degenerative disease. In case of diseased cells possessing mutation, the mutation is first corrected in order to be able to form normal iPSCs, and then these iPSCs are differentiated into specific cell types by providing specific conditions essential for the development of those cells. These repaired cells can then be transplanted into the body of the organism from which the cells for the generation of iPSCs were isolated.

A major difficulty in the application of iPSCs for regenerative medicine is the delivery of reprogramming factors. piggyBac transposon-based approach to generate integration-free iPSCs is highly efficient and most promising [74,75,76] because piggyBac excises without a footprint, leaving the iPSC genome without any genetic alteration and with hallmark pluripotency markers. Though the existence of piggyBac-like elements in the human genome [77] raises the question of safety concerns. Another concern shared by both non-viral and viral vectors, is the integration site specificity and its counterpart insertional mutagenesis. Sequence-specific DNA-binding proteins such as homing nucleases, zinc finger proteins (ZFPs), transcription activator-like effector nucleases (TALENs) and Cas9 of the CRISPR/Cas system have been adapted to introduce gene editing which in turn might cause safety issues, not fully investigated so far [78].

The complete list of conditions, which could be treated with iPSCs in the future, would be beyond the scope of this review but here are a number of conditions that can potentially be treated with this technology. Hematopoietic disorders, liver damage and Spinal cord injury could be treated by the generation of specific cells with the help of iPSCs [79,80,81].

Of late, researchers have been assembling data on the use of iPSC for ex vivo blood expansion of various blood components. They can be used for the generation of Red Blood Cells (RBCs), which could be utilized for generating blood for the purpose of treatment of various damages/diseases prevalent in world. There are several techniques by which we can use iPSCs for the production of RBCs [82].

iPSCs can also be used for the generation of various cells which can help in the repair of many tissues, for example, cardiomyocytes for repairing heart valves, vessels and ischemic tissues, but there are limitations like post treatment side effects, safe delivery and protocol standardization to produce big volumes of pure, good quality cells. These hurdles once overcome, offer great opportunities for iPSC applications for generating cardiovascular cells and studying corresponding diseases [83,84,85].

Another plus is iPSCs can be derived from immune cells as equally as they can redifferentiate into specific immune cell types for clinical immunotherapy. Dedritic cells (DCs) are the most potent antigen-presenting cells. Thus, DC-based cellular vaccination provides a powerful means for immunotherapy, especially against cancer. In addition, antigen-specific negative regulation of immune response by DCs is considered to be a promising approach to treat autoimmune diseases and in transplant medicine. iPSCs may be an ideal source for DCs that can broaden their immunotherapeutic applicability. In 2009, Senju *et al*. [86] reported that the iPSC-derived DCs are functional in that they effectively processed and presented antigens, stimulated T cells, and produced cytokines.

#### 4.3.1. First ever Clinical Trial

At Riken Centre for Developmental Biology, Japan, the Takahashi team clinical study intended to examine the safety of a human RPE cell product made from each patients’ own iPSCs had been going on. Takahashi’s team conducted safety trials in both monkeys and mice before the first ever iPSC clinical study in humans [87,88]. The animal tests revealed that iPSCs were not rejected and did not cause cancerous growth. Japan health-ministry committee assessed researchers’ safety tests and cleared the team to begin the experimental procedure in September 2014 [89,90]. In an astounding feat of swift clinical translation, Takahashi’s team transplanted its first macular degeneration patient on 12 September 2014 (under GMP conditions) only 7 years after hiPSCs were first ever published [91]. The usual timeline for such translation would be 20 years. Takahashi had reprogrammed some cells from the 70-year old woman patient’s skin to produce iPSCs. She then coaxed those cells to turn on the retinal genes, differentiate into RPE cells and grow into a sheet for implantation. After surgically removing spurious blood vessels and damaged tissue from the affected eye, this cell sheet was transplanted to the damaged area of the retina. The researchers anticipate the cell growth at the transplanted site, which in turn will expectantly repair the pigment epithelium. The procedure has apparently progressed satisfactorily and has been able to stop patient’s vision from further deterioration; however, the experimental procedure was not performed on further individuals when genetic mutations were identified in the iPSCs [92]. In 2015, Zhao *et al.* [93] demonstrated in humanized mice that an immune response is mounted against iPSC-derived smooth muscle cells but not iPSC-derived RPE, suggesting that autologous iPSC derivatives may have differential immunogenicity.

#### 4.3.2. Ongoing Clinical Trials

Phase I–II clinical trials using hESC-derived insulin-producing progenitor β-cells as a subcutaneous flat encapsulated product, a little smaller than a credit card, is underway by the company ViaCyte (San Diego, CA, USA) to treat type 1 diabetes patients [94].

Recent studies have shown that hESC-derived cardiomyocytes and cardiovascular progenitors are able to halt the deterioration of cardiac function and improve experimentally induced diminished heart function in rodent and monkey models [95,96]. Moreover, in a monkey myocardial infarct model, intramyocardial delivery of 1 billion hESC-derived cardiomyocytes revealed extensive remuscularization; although, potential complications due to ventricular arrhythmias are still a concern.

A 68-year-old patient suffering from severe heart failure was surgically grafted (Paris, France) onto the infarcted area with hESC-derived cardiomyocyte progenitors expressing the early cardiac transcription factor insulin gene enhancer Isl-1 and stage-specific embryonic antigen (SSEA)-1, which are cell markers for cardiac progenitors. The cardiac progenitors were then embedded into a fibrin scaffold to enable the integration of grafted cells from the patch into damaged heart tissue [97]. A coronary artery bypass surgery was performed concomitantly in a non-infarcted area followed by uncomplicated post-operative recovery. The patient symptomatically improved after 3 months, although this may have been owing to revascularization resulting from the coronary bypass surgery. Notably, contractility was echocardiographically observed in the previously akinetic heart patch area, and no arrhythmias, tumour formation, or immunosuppression-related adverse effects were evident [97,98].

Asteria Biotherapeutics (California, USA) with the funding from California Institute for Regenerative Medicine (CIRM) started phase I–II clinical trial on patients suffering from spinal cord injury by injecting them with hESC-derived oligodendrocyte progenitors in 2015. They started the trial with a dose escalation study where the first group of three patients received two million cells. Apparently, the dosage has been safe and one patient has shown encouraging results. At present, they are enrolling patients for the 10 million cell dose trial, which if successful, is to be followed by 20 million cell dose [99].

In Australia, there is a recent announcement by International Stem Cell Corporation (USA) that will begin phase-I clinical trial to treat Parkinson’s disease by injecting 12 sufferers with parthenogenetic (pluripotent human stem cells from unfertilized oocytes) neural stem cells and observe them for 1 year [100].

### 4.4. Development of iPSC Library

One of the major challenges of the use of ESCs in cell therapies is an immune-mediated rejection after transplantation. Today, this problem can be overcome by direct reprogramming of patients’ somatic cells and by creating an iPSC bank consisting of various human leukocyte antigens (HLA) types thus providing therapeutic tool for the patients needing cell transplantation, free from immune-mediated rejection [101,102]. In addition, each cell line should contain detailed genetic and epigenetic profiles and differentiation potential ‘‘score cards’’ [103,104]. Two works reported that the establishment of 50 unique stem cells lines, having homozygous alleles of the 3 HLA loci (A, B, and DR), would cover 90% of the Japanese population with a faultless match of these loci [105,106]. Considering that derivation and testing iPSCs tailored for individual patients is a time consuming process (about 6 months for each cell line) and costs tens of thousands of dollars, it is quite necessary for cell therapies and regenerative medicine to establish iPSC banks with a sufficient collection of HLA types, thus avoiding additional expenses which are required for iPSC production for each individual patient.

The Center for iPSC Research and Application (CiRA), Kyoto University, Japan under the guidance of Shinya Yamanaka, in collaboration with Kyoto University Hospital, Japan embarked on a project of building the Clinical iPS Cell Bank of Kyoto (CiBK) with lines from approximately top 100 haplotypes donors providing coverage for about 90% of the population in Japan. Yamanaka’s project has an advantage because genetic diversity in Japan is relatively low; elsewhere, therapeutic banks would have to be larger and costlier. Using blood from Japan’s eight cord-blood banks will also make the task easier as the banks hold some 29,000 already HLA-characterized samples. The establishment of iPSCs is to be done at clinical grade, from peripheral blood or umbilical cord blood, in the Cell Processing Center that is part of CiRA [107].

Most iPSC banks outside Japan specialize in cells from people with diseases, for use in research rather than treatment. The California Institute for Regenerative Medicine (CIRM) in San Francisco, for example, plans to bank some 3000 cell lines for distribution to researchers [108].

Recently, the human iPSC initiative (HipSci) reported the establishment of a high-content platform for phenotypic analysis of human iPSC lines [109]. In the described assay, cells are dissociated and seeded as single cells onto 96-well plates coated with fibronectin at three different concentrations. This method allows assessment of cell number, proliferation, morphology and intercellular adhesion. Altogether, this strategy delivers robust quantification of phenotypic diversity within complex cell populations facilitating future identification of the genetic, biological and technical determinants of variance. Approaches such this can be used to benchmark iPSCs from multiple donors and create novel platforms that can readily be tailored for disease modelling and drug discovery. This platform could make the enormous task of developing iPSC library on a world-scale to cater the diverse population less cumbersome.

## 5. Challenges

### 5.1. Choosing an Appropriate Somatic Cell Type

Choosing a suitable cell type for reprogramming is of critical concern for future autologous patient-specific iPSC production and clinical therapy. The ideal cell source to be isolated from the patients and used for reprogramming must meet the criteria of easy accessibility with minimal risk procedures, availability in large quantities, relatively high reprogramming efficiency, and fast iPS cell derivation speed. Moad *et al.* used human prostate and urinary tract cells for the formation of iPSCs and further for studying the mechanisms that regulate the differentiation of prostate and urinary tract cells. With their study, they concluded that iPSCs generated from prostate and urinary tract had better efficiency of differentiation to cells of prostate and urinary tract as compared to iPSCs derived from skin fibroblasts which showed that organ of origin plays an important role in terms of efficiency of differentiation [110]. Table 1 shows a comparison of the different cell origins that have been used for reprogramming.

Another study has shown that persistent donor cell gene expression memory in hiPS cell lines can contribute significantly to the differences among hiPSCs and human ESCs, and adds to the incompleteness of reprogramming [118]. Hence, the optimal cell source for generating patient-specific iPSCs should be carefully selected on a patient-specific basis, when it proves possible to evaluate specific conditions of individual patients in the future [111].

### 5.2. Variability and Heterogeneity

A vital factor of consideration in iPSCs generation is a concern raised by studies performed on genetic and epigenetic variations in iPSCs and how these variations compromise the utility of iPSCs and undermine their accountability in downstream applications [119]. These variations exist between iPSC lines, between iPSC and ESC lines, between different passages of the same iPSC line, and even between different populations at a specific passage of the same iPSC line. These variations occur due to different sources; some of the variations may be inherited from donor somatic cells, induced or selected by the reprogramming process, or accumulated during culturing; others may simply reflect the innate genetic and epigenetic stability of the pluripotent state of iPSCs. Although each variation is not relevant to the functionality of iPSCs, certain variations may change the properties of iPSCs and their derivatives. For example, the variations may alter the differentiation potential of iPSCs, cause phenotypic changes in iPSC-derived somatic cells, or increase the tumorigenicity or immunogenicity of iPSCs and their derivatives. One of the causes for the varied differentiation capacity is source cell memory, which biases iPSC differentiation into the source cell lineage [120,121,122]. These adverse changes directly affect the utility of iPSCs. Accumulating evidence also indicates that epigenetic mechanisms not only play important roles in the iPSC generation process, but also affect the properties of reprogrammed iPSCs [123]. Extended passage of some iPSC clones in culture did not improve their epigenetic resemblance to ESCs, implying that some human iPSCs retain a residual ‘epigenetic memory’ of their tissue of origin [121]. In a study performed by Deng *et al.* [124] iPSCs appeared to display more methylation than ESCs. Some studies have identified hotspots or sets of “signature” genes in hiPSCs whose DNA methylation and transcription statuses are clearly different from that of hESCs [124,125,126,127]. However, another comprehensive study concluded that the variations of DNA methylation between hESCs and hiPSC lines are not greater than those between different hESC lines [103]. These studies question the biological consequences of such deviations in pattern for future therapeutic applications of iPSCs and demand more in-depth research to understand the reprogramming process thoroughly.

Despite the common ability of hiPSCs and hESCs to differentiate into all 3 germ layers, a recent single cell analyses revealed much more heterogeneity in gene expression levels in iPSCs than in ESCs [128]. This suggests exercising caution before assuming that hiPSCs occupy a pluripotent state equivalent to that of hESCs, especially when producing differentiated cells for regenerative therapeutic aims.

### 5.3. Validation of Pre-Clinical iPSC Therapies

As discussed above, Takahashi’s team successfully transplanted first patient with reprogrammed iPSCs as RPE cells in September 2014 after safety trials on monkeys and mice both. The safety and therapeutic applications of these cells must be meticulously tested in appropriate animal models before advancing to any other clinical trial as well. We need to study minimum number of undifferentiated iPSCs that can cause teratoma or teratocarcinoma in autologous transplantation animal models, as residual undifferentiated cells may still remain after differentiation into specific cell lineages and may lead to tumorigenicity after transplantation.

Another concern is oncogenic transgene integration and insertional mutagenesis may be associated with many of the currently established iPS cell lines, the questions of whether iPSCs generated with different reprogramming technologies as well as their derivatives can induce cancer in the host also need to be strictly evaluated. Even with improvements in the virus-free and transgene-free reprogramming technologies, the cancer-causing possibility of the derived “safe” iPSCs/derivatives still needs to be assessed in animal models before using them clinically for regenerative therapy. In addition, iPS cell therapies need to be validated not only in small animals (mice and rats) but also in large animal models that are anatomically and physiologically more similar to humans. Both monkey [129] and pig [130,131,132] iPS cells have been generated, providing excellent models for transplantation studies. Although the thorough pre-clinical evaluation of iPS cells would be arduous, it is absolutely essential for the future.

### 5.4. Regulatory and Commercial Hurdles

Given the many probable risks of applying autologous iPS cell treatment to humans, iPSC therapies may encounter strict regulatory restrictions in some parts of the world, including in Europe, United Kingdom and the United States. In addition, clinical GMP-compliant vectors for iPSC reprogramming are currently unavailable. Another issue that may impede the clinical translation is the financial feasibility of producing individualized iPSC therapeutic products. The viability of a business model for patient-specific iPS treatment is still unknown. It may well be the case that few if any pharmaceutical companies will be able to produce cost-effective individualized iPSC products tailored for a single patient at a time. On the other hand, we cannot deny the exceptional potential of iPS therapies. For instance, if researchers are able to solve the immune tolerance problem [133] then one can expect allogeneic transplantation of iPSC products. These cells will need to be made in GMP-compliant, large-scale production for it to be commercially viable [134], and the individual needs or profiles of patients will need to be easily assessed to allow matching and wide distribution. For example, an optimized dose for hematopoietic stem cell transplantation for a 70-kg adult patient was suggested to contain 4.2 × 10^8^ to 5.6 × 10^8^ CD34^+^ cells [135]. Production of a clinically relevant quantity of hiPSCs and/or their progenies for specific applications, sometimes considered as ~1 to 2 billion [136], in a chemically defined condition by robust, reproducible and economic methods remains a major challenge for advancing hiPSC technology from the bench to the clinic [32]. The cell manufacturing process will need to adhere to additional country-specific guidelines given that iPSC-derived cells may be distributed internationally, even to countries where regulations are yet to be formulated.

## 6. Future of iPSCs in Regenerative Dentistry

### 6.1. Gingiva as an iPSC Source

**Figure 3:**
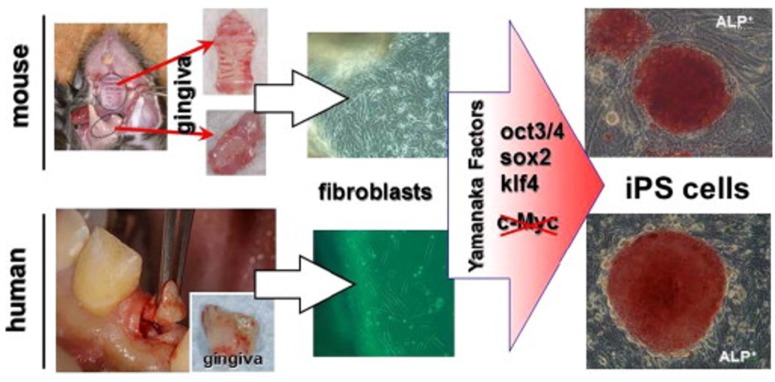
Generation of iPS cells from gingiva [138]. Gingival tissues from adult mice and patients (resected during dental implant surgery) can be used for iPS cell generation. Isolated gingival fibroblasts were easily reprogrammed by the transduction of three factors (Oct3/4, Sox2, and Klf4) without the c-Myc oncogene. Red iPS colonies in the figure show robust staining for alkaline phosphatase (ALP), which is associated with undifferentiated pluripotent stem cells. This figure is reproduced from the study by Egusa *et al.* [137] under open-access license policies.

Oral gingiva is often resected during general dental procedures and considered as biomedical waste, is an easily obtainable tissue, and cells can be isolated from patients with minimal discomfort. iPSCs can be easily generated from adult mouse or human gingival fibroblasts by transduction of three Yamanaka factors (Oct3/4, Sox2 and Klf4) without c-Myc and omission of c-Myc oncogen transduction has actually been shown to result in more specific iPSC generation [137] (Figure 3). Furthermore, higher reprogramming efficiency in iPSCs was observed by using gingival fibroblasts compared to conventionally used skin fibroblasts, perhaps because of their high proliferative capability [137].

The gingiva consists of highly vascularized connective tissue under a thin keratinocyte layer, and gingival fibroblasts, which are phenotypically different from other fibroblasts, are the main constituents of this connective tissue [139]. Clinical and experimental findings in patients and animal models have consistently demonstrated that oral mucosa, including gingival tissue, has enhanced wound healing capability compare to skin [140,141], even though both tissue types share similar healing processes and sequences. Another advantage is that gingival fibroblasts contain fibroblastic stem cell population [142]. The multipotent subpopulation and high regenerative capability of gingival fibroblasts may represent a progenitor-like state that could enhance the iPSC reprogramming efficiency of these cells comparatively as less reprogramming would be required to reach the anticipated pluripotent state. Moreover, gingival fibroblasts expand and proliferate well and easily on tissue culture plates [143]; hence primary gingival fibroblast cultures could be established quite effortlessly.

These cells are outstandingly suitable for iPSC-related clinical applications because of their accessibility, ease of culture and reprogramming competency. It may be convenient to apply these cells to achieve the regeneration of complicated tissues/organs, such as the tooth and salivary gland, which are formed through the interaction of epithelial and mesenchymal tissues during organogenesis.

### 6.2. Tooth Bioengineering

**Figure 4:**
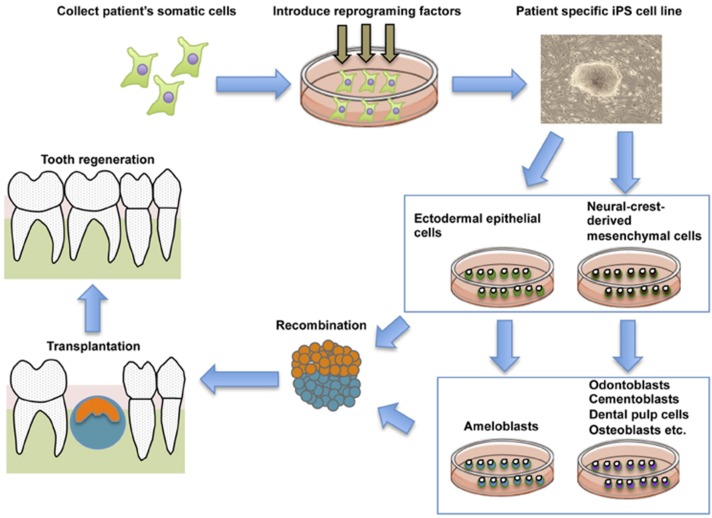
General schematic representation of the current strategy for whole tooth regeneration using iPSs. The patient’s somatic cells are harvested. Reprogramming conditions/factors are introduced to induce self-renewal and pluripotency, and patient-specific iPSCs are established. iPSCs are induced to form ectodermal epithelial cells and neural crest-derived mesenchymal cells, and they are further induced to form odontogenic cells *in vitro*. The two cell populations are combined by direct contact, mimicking the *in vitro* arrangement. Interaction of these cells leads to formation of a nearly-stage tooth germ. Once transplanted into the mouth, the recombinants develop and lead to functional recovery from tooth loss. This figure is reproduced from Otsu *et al.* [148] under open-access license policies.

Reciprocal interactions between dental mesenchymal cells derived from Neural Crest (NC) and dental epithelial cells derived from ectodermal epithelium controls tooth development [144,145]. Epithelial-mesenchymal interactions also control the terminal differentiation of odontoblasts and ameloblasts [146,147]. Thus, as a new strategy for tooth regeneration, it is speculated that ectodermal epithelial cells and NC cells induced from iPS cells could be the optimal cell source for the whole tooth regeneration (Figure 4).

### 6.3. Periodontal Ligament Regeneration

A report by Oshima *et al.* in 2014 [149] opened the way for generation and regeneration of periodontal tissue by using Dental Follicle (DF) cells from tooth germ at temporally-limited stage of development, while it was restricted to murine specimen. Therefore considering clinical practice in patients for structuring human periodontal tissue with much larger size, researchers need to clarify the characteristics of DF cells at ED 18.5, comparing with those at other stages or periodontal ligament stem cells (PDLSCs), and how they can obtain such DF-like cells from iPSCs because the quantity of PDLSCs localized in periodontal ligament tissue is too small to use in clinical use while PDLSCs are naturally eligible. In addition, which bioactive scaffolds and multifunctional signal molecules support the differentiation of these stem cells also remains to be determined. Sufficient assessment of outcomes from integration of these elements from various aspects is needed for restoration of periodontal function [150].

## 7. Concluding Remarks and Future Perspectives

During the past decade since its inception, iPSC technology has shown great potential for clinical applications and disease modelling [151,152]. iPSCs are an exceptional source of cells for regenerative medicine as they demonstrate indefinite proliferation, obtainability and plasticity to differentiate into other cell types. There is a minimal risk of immunorejection and no ethical baggage. This potential is further strengthened by combining iPSC technology with genome engineering [153], which allows the correction of mutations in patient-derived iPSCs and use it to our advantage for personalized treatment and disease modelling, as well as modification of reporter lines to facilitate differentiation towards specific cell types. These properties successfully reduce expenditure and risk of clinical trials, and also provide phenotypically consistent cell lines capable of predicting cytotoxic and therapeutic responses of drugs. The field is full of great promises but, as discussed in this review, is still in its infancy and many obstacles remain to be overcome.

Despite the advantages, iPSC technology still faces many pitfalls such as low efficiency and high variability. Donor cell source and vector type still need major contemplation and reprogramming method still needs optimization. Our ultimate goal is to demonstrate therapeutic potential of iPSCs in clinics and it can only be achieved by generating hiPSCs affordably in GMP-compliant manner. The many clinical trials that are underway give us assurance of the coming revolution in near future and the present information available is satisfactorily promising.

## Figures and Tables

**Table 1:** Comparison of different cell origins used for reprogramming [111].

Cell Source	Derivation	*In vitro* Expansion	Reprogramming Efficiency (4 factor)	Reprogramming Speed	Reprogramming Factors	References
Skin Fibroblasts	Skin Biopsy	Yes	~0.01% (Adult cells)	>21 days	OSKM, OSK, OSNL	[5]
Keratinocytes	Skin Biopsy	Yes	~1% (neonatal and juvenile cells)	>10days	OSKM, OSK	[112]
CD34 Blood Cells	Peripheral Blood undergo G-CSF stimulation	No	~0.01-0.02% (Adult Cells)	>14days	OSKM	[22]
Adipose Stem Cells	Lipoaspiration	No	~0.2% (Adult Cells)	>13–14 days	OSKM	[113]
Melanocytes	Skin Biopsy	Yes	~0.05% (not known)	>10 days	OSKM, OKM	[114]
Cord Blood Cells	Collected at birth from cord cells	No	~0.01% (neonatal cells)	>12–15 days	OSKM, OSNL, OSK, OS	[115,116]
Neural Stem Cells	NA	Yes	0.004% (1 Factor, Fetal cells)	>7–8 Weeks (1Factor)	OK, O	[117]

O: Oct4, S:Sox2, K: Klf4, M: c-MYC, N: Nanog, L: Lin-28, NA: Not Applicable.

## Abbreviations

The following abbreviations are used in this manuscript:

iPSCs	induced pluripotent stem cells
hiPSCs	Human induced pluripotent stem cells
OSKM	Oct4, Sox2, Klf4, and c-Myc
hESCs	human embryonic stem cells
ESC	Embryonic Stem Cells
SOX2	the sex determining region Y-box2
Klf4	Kruppel-like factor 4
c-Myc	myelocytomatosis oncogene
GMP	Good Manufacturing Practice
ECM	Extracellular matrix
5hmc	5-hydroxymethylcytosine
RPE	retinal pigment epithelial
RBCs	Red Blood Cells
DCs	Dedritic cells
SSEA	stage-specific embryonic antigen
CIRM	California Institute for Regenerative Medicine
HLA	human leukocyte antigens
CiRA	Center for iPSC Research and Application
CiBK	Clinical iPS Cell Bank of Kyoto
NC	Neural Crest
DF	Dental Follicle
PDLSCs	periodontal ligament stem cells
